# Dry Matter Yield Stability Analysis of Maize Genotypes Grown in Al Toxic and Optimum Controlled Environments

**DOI:** 10.3390/plants11212939

**Published:** 2022-11-01

**Authors:** Rutendo M. Zishiri, Charles S. Mutengwa, Aleck Kondwakwenda

**Affiliations:** Department of Agronomy, University of Fort Hare, Private Bag X1314, Alice 5700, South Africa

**Keywords:** discriminating ability, plant breeding, AMMI, GGE biplot, seedlings, inbred lines

## Abstract

Breeding for Al tolerance is the most sustainable strategy to reduce yield losses caused by Al toxicity in plants. The use of rapid, cheap and reliable testing methods and environments enables breeders to make quick selection decisions. The objectives of this study were to (i) identify high dry matter yielding and stable quality protein maize (QPM) lines grown under Al toxic and optimum conditions and (ii) compare the discriminating power of laboratory- and greenhouse-based testing environments. A total of 75 tropical QPM inbred lines were tested at seedling stage for dry matter yield and stability under optimum and Al toxic growing conditions across six laboratory- and greenhouse-based environments. The nutrient solution method was used for the laboratory trials, while the soil bioassay method was used for the greenhouse trials. A yield loss of 55% due to Al toxicity was observed, confirming the adverse effects of Al toxicity on maize productivity. The ANOVA revealed the presence of genetic variation among the set of genotypes used in this study, which can be exploited through plant breeding. Seventeen stable and high-yielding lines were identified and recommended. Greenhouse-based environments were more discriminating than laboratory environments. Therefore, we concluded that greenhouse environments are more informative than laboratory environments when testing genotypes for Al tolerance.

## 1. Introduction

Low pH or acidic soils are one of the major limitations to maize (*Zea mays* L.) productivity in the tropics. Acidic soils are widely distributed in tropical and subtropical areas, constituting about 50% of the world’s potentially arable soils. They are found in approximately 20% of the world’s maize mega environments [1,2]. Acidic soil conditions enhance the solubility of aluminum (Al) and other metals, which leads to toxicity in maize and other sensitive crops [2,3].

Aluminum toxicity adversely affects plant growth and development by inhibiting cell division and the expansion of root meristems, causing stunted root elongation [4]. This reduces the capacity of plants to uptake nutrients and water, exposing plants to drought, which in turn causes poor dry matter accumulation and yield loss. Liming provides a quick and short-term solution for soil acidity and Al toxicity, but it is beyond the reach of most farmers in the developing world. Genetically improving maize cultivars to be tolerant to Al toxicity through plant breeding is a sustainable strategy to curb the effect of Al toxicity on maize production in acid soils. Great efforts have been made so far in Africa towards breeding maize cultivars that are tolerant to various abiotic and biotic stresses, including drought stress [5,6], low soil nitrogen [7], various diseases and pests [8,9] and other stresses [10]. However, more work is still required to develop maize cultivars tolerant to Al toxicity.

Testing and selecting stable parental lines that combine Al tolerance, high yield potential and stability are the initial critical steps in breeding for Al tolerance in plants. Testing maize for Al tolerance can be carried out in the field, laboratory or greenhouse. Although field testing has proved to be the most effective in testing maize genotypes for Al tolerance, it is very expensive and time-consuming [11,12]. Laboratory- or greenhouse-based testing techniques offer cheap, easy, and quick alternatives to screening large sets of genotypes for Al tolerance [13,14]. The other advantage of laboratory- and greenhouse-based testing methods is that they are very amenable to seedling-stage testing techniques. Seedling stage testing allows breeders to make selections at an early stage of plant growth, thereby increasing genetic gain by shortening the breeding cycle. Thus, seedling stage selection supports speed breeding, which is one of the effective modern breeding technology [15]. In addition, it has been proven that laboratory- and greenhouse-based testing techniques give results that are positively correlated with those obtained using field testing methods [16,17,18].

The nutrient solution method is the most used laboratory-based technique, while the soil bioassay, also known as the pot-based method, is the most popular greenhouse-based technique for testing plant seedlings for tolerance to Al toxicity [13,14]. These two methods provide adequate Al stress to the plants, allowing the effective preliminary testing of many genotypes in a small area and consequently reducing the number of promising genotypes to be taken to the field for further analysis. However, given the differences between the laboratory and greenhouse growing conditions, it is important to determine how maize genotypes perform in and across the two environments. Logically, genotypes that can perform well in the laboratory and greenhouse are likely to be stable performers, and the chances that they will excel in the field are high. Furthermore, genotypes that exhibit high and stable performance across both Al toxic and optimum conditions are desirable for a breeding program, as they allow breeders to develop cultivars that can thrive on both acidic and non-acidic soils. However, the discriminating ability of laboratory- and greenhouse-based methods and environments among maize genotypes has never been compared. Therefore, it is important to find out which one of the two-testing environments is the most effective in discriminating among maize genotypes for Al tolerance or susceptibility.

In this study, the additive main effect and multiplicative interaction (AMMI) model was used to assess the performance and stability of maize lines across laboratory- and greenhouse-based test environments [19]. One of the strengths of AMMI analysis is that it provides AMMI stability values (ASV), which help identify stable genotypes across environments. Good stability is indicated by a lower ASV, while a higher ASV reflects the poor stability of a genotype [19]. However, AMMI analysis is ineffective in showing the discriminating power of test environments compared to the genotype plus genotype by environment interaction (GGE) biplot [20]. Therefore, the GGE biplot was employed in this study to compare the capacities of laboratory and greenhouse environments to discriminate among genotypes [21].

This study aimed to identify high dry matter yielding and stable quality protein maize (QPM) inbred lines at the seedlings stage grown under Al toxic and optimum laboratory- and greenhouse-based environments. The study also sought to compare the discriminating ability of laboratory- and greenhouse-based Al tolerance testing environments. The study is a preliminary dry matter yield stability analysis of elite tropical QPM maize inbred lines for Al tolerance. The lines are an important part of the germplasm used in the QPM breeding program at the University of Fort Hare (UFH). The hypotheses of the study were that there are (i) no stable genotypes in terms of dry performance across Al toxic and optimum environments among the QPM lines available at the UFH’s breeding program and (ii) no differences between the laboratory- and greenhouse-based environments in terms of discriminating maize genotypes for Al tolerance.

## 2. Results

### 2.1. Analysis of Variance

The combined ANOVA for the dry matter yield of the 75 QPM genotypes evaluated across six environments is shown in Table 1. Highly significant differences (*p* < 0.001) were observed for genotype (G), environment (E) and genotype × environment interactions (G × E). This prompted further analysis of G × E using AMMI and GGE biplots, as described in the following section. The genotype main effect contributed 6.08% to the total sum of squares, while the environment and genotype by environment interaction had 82.82% and 11.08%, respectively.

### 2.2. AMMI and GGE Biplots

The AMMI ANOVA for the dry matter yield of the 75 QPM inbred lines tested in six environments is presented in Table 2. The genotype, environment and genotype × environment interaction (GEI) effects were highly significant (*p* < 0.001). The GEI effect accounted for 11.08% of the total variation, which was further partitioned into two highly significant (*p* < 0.001) interaction principal components axes (IPCA 1 and IPCA 2).

**Figure 1:**
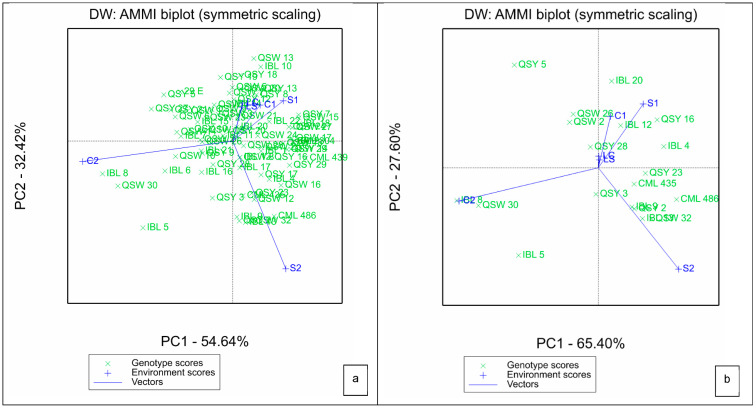
Additive main effects and multiplicative interaction biplot for IPCA 1 and IPCA 2 scores of (**a**) all the 75 QPM genotypes and (**b**) 20 highest yielding genotypes (**b**) and six environments. S1: Al toxic environment under soil bioassay conditions in the greenhouse 1; S2: Al toxic environment under soil bioassay conditions in the greenhouse 2; C1: control environment under soil bioassay conditions in the greenhouse 1; C2: control environment under soil bioassay conditions in the greenhouse 2; LC: control environment under nutrient solution conditions, LS: Al toxic environment under nutrient solution conditions.

For the AMMI, the two IPCAs explained a total of 87.43% of G × E variation, with IPCA1 contributing 54.64% and IPCA2 providing 32.46% to the biplot (Figure 1a). Genotypes that were located at or closer to the point of origin are considered to be broadly adopted. Line QSY 28 was the most broadly adapted, as it is located closer to the point of origin (Figure 1b). The length of the vector of an environment from the biplot origin is proportional to the amount of G × E exhibited by that environment. Thus, environments with longer vectors possess strong interactive forces, while those with shorter vectors produce weak interactive forces. In this regard, environments S1, C2 and S2 had stronger interactive forces than LS, LC and C1. Generally, greenhouse environments had stronger interactive forces than laboratory environments, which had the weakest interactive forces (Figure 1a,b). Genotypes closer to the environment scores showed high interactive behavior with the respective environments.

**Figure 2:**
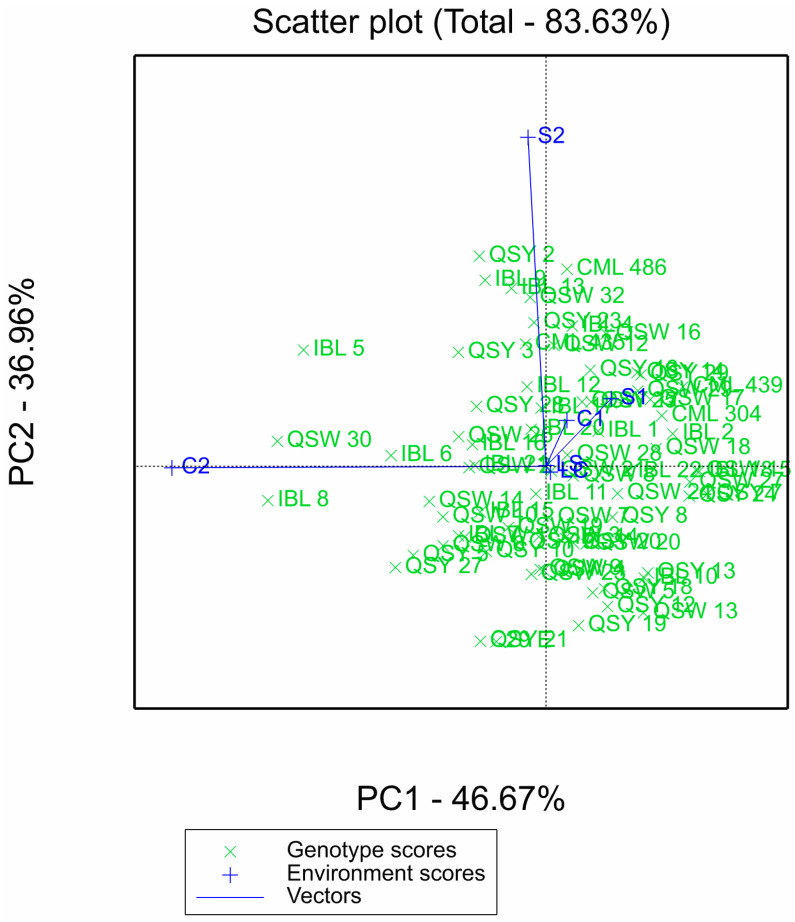
GGE biplot of the 75 QPM inbred lines evaluated across six laboratory and greenhouse environments. S1: Al toxic environment under soil bioassay conditions in the greenhouse 1; S2: Al toxic environment under soil bioassay conditions in the greenhouse 2; C1: control environment under soil bioassay conditions in the greenhouse 1; C2: control environment under soil bioassay conditions in the greenhouse 2; LC: control environment under nutrient solution conditions, LS: Al toxic environment under nutrient solution conditions.

For the GGE biplot, the two principal components contributed a total of 83.63%: PCA1 had 46.67%, while PCA2 had 36.96% (Figure 2). The length of the environmental vectors is proportional to their standard deviation, which is a measure of the discriminating ability of the environments. The environments with longer vectors are more discriminating than those with shorter vectors. Environments S2 and C2 had the longest vectors, followed by S1 and C1, with laboratory environments (LS and LC) having the shortest vectors (Figure 2).

### 2.3. Mean Dry Matter Yield, ASV and Top Four Genotypes across Environments

The genotypes’ mean dry matter yield performance across the six environments were non-consistent. The performance of the top ten, top two checks and bottom five yielding genotypes across the six environments is presented in Table 3, while the performance of all 75 genotypes is presented in Appendix A (Table A1). Thus, the ranking of genotypes changed from one environment to another. Higher dry matter yields were observed in optimum environments as compared to Al toxic environments with a 55% mean yield difference. The grand mean weight across the six environments was 19.1 g. Thirty-six experimental lines yielded above the grand mean. Experimental inbred line QSY 2 was the highest yielder with 23.9 g, followed by experimental lines IBL 5 and IBL 9 with 23.2 and 23.0 g, respectively. The highest yielding check was CML 486 with 21.9 g. Five experimental lines (QSY 2, IBL 5, IBL 9, QSW 30 and QSW 26) yielded above the top yielding check. The first optimum greenhouse environment (C1) had the highest mean biomass yield of 37.8 g, followed by optimum greenhouse environment (C2) with 35.7 g. The laboratory Al toxic environment (LS) had the lowest biomass yield of 0.1 g.

The AMMI stability values for genotypes ranged from 0.26 (QSY 20) to 4.816 (IBL 8). The mean ASV was 1.64. The five genotypes with the least ASV were QSY 20, IBL 20, IBL 11, QSW 28 and QSW 8 with values of 0.26, 0.34, 0.37, 0.41 and 0.44, respectively (Table 4). Seventeen lines had a dry matter yield above the grand mean (19.1 g) and ASV lower than the grand mean of 1.64. These lines were QSW 26, QSY 3, IBL 20, QSY 23, IBL4, IBL12, QSY 28, QSY16, QSW2, QSW 25, QSW 21, QSW 12, QSW 7, IBL17, IBL 21, IBL 15 and IBL 16.

The top four selections from each of the six environments are shown in Table 4. Experimental line IBL 20 appeared in the top four in four of the six environments, which are LS, LC, S1 and C1, whilst experimental line QSW7 appeared in the top four in environments LS, LC and C1. Lines QSW 26, QSY 5 and QSW 26 appeared in the top four in two environments each.

## 3. Discussion

Both general and AMMI ANOVAs revealed considerable variation among the 75 QPM genotypes and the six laboratory and greenhouse environments. Genotype (G), environment (E) and genotype by environment interaction (G × E) were highly significant (*p* < 0.001). The significance of the genotype main effect implies that there is genetic variability among the set of genotypes used in this study. Hence, there is an opportunity for selection and breeding for Al tolerance using the set of lines understudy. This result confirms what was reported by [15] in their study using the relative root length (RRL) and hematoxylin staining (HS) to classify a similar set of genotypes into Al tolerant and sensitive. Using the RRL method, they found out that 94.7% of the lines were Al tolerant, while using the HS technique, they reported 77.9% of the genotypes to be tolerant. However, the fact that only 6.08% of the total variation was attributed to genotype indicates a need to introduce more sources of variation from external sources or through artificial mutation. Genetic variability for Al tolerance has previously been reported in maize inbred lines, hybrids and open-pollinated varieties by other researchers [12,13,22].

Significant G × E indicates that genotype performance was inconsistent across the six environments. Thus, the selection of the best lines cannot be based on ranking but on whether they are broadly or specifically adapted to environments [19]. However, the focus of this study was to identify lines that combine high dry matter yield and stability across Al stressed and optimum laboratory- and greenhouse-based environments. Genotypes with low ASV are considered stable, while those with high ASV are less stable. In this study, seventeen experimental lines had the desired combination of high dry matter yield above the grand mean (19.1) and ASV less than the grand mean (1.64). These are QSW 26, QSY 3, IBL 20, QSY 23, IBL4, IBL12, QSY 28, QSY16, QSW2, QSW 25, QSW 21, QSW 12, QSW 7, IBL17, IBL 21, IBL 15 and IBL 16. Such lines are potential donors of Al tolerance genes that can be exploited through plant breeding. Therefore, they are recommended for further field-based testing.

The other objective of this study was to compare the discriminating ability of laboratory- and greenhouse-based Al tolerance testing environments. The discriminative power of an environment is directly proportional to its vector length on the GGE biplot. Both GGE and AMMI biplots revealed that the greenhouse-based environments (S1, S2, C1 and C2) were more discriminating and had more interactive forces than the laboratory ones (LS and LC). Therefore, we conclude that testing maize genotypes for Al tolerance in the greenhouse environment is more informative and effective than using the laboratory environment. This could be attributed to the fact that greenhouse conditions and soil bioassays provided better growing conditions to genotypes so that they could express their performance compared to laboratory conditions and nutrient solutions. This result agrees with [23], who found soil bioassays more efficient than nutrient solution in discriminating genotypes for Al toxicity. The use of controlled environments such as greenhouses is becoming increasingly important in plant breeding, mainly due to their suitability for modern plant breeding techniques such as speed breeding [15,24] and marker-assisted breeding [25]. Furthermore, they allow breeders to implement early growth stage selection, which helps achieve high genetic gain and quick varietal turnover. The application of modern breeding techniques has proven crucial for achieving food security [26].

The fact that the environment main effect was significant and had the largest contribution (82.82%) to the total variation underlines the varying effects that the different environments had on the genotype’s performance. This could be attributed to the huge differences between laboratory and greenhouse growing conditions and between optimum and Al-stressed environments. The large contribution of testing environments has been reported in other studies on abiotic stress tolerant stability analysis [27,28]. In their study, [29] reported an environmental contribution of 80%, which is almost equal to our findings.

A yield loss of 55% due to Al toxicity was observed in this study, which confirms the negative effect of Al toxicity on maize productivity. A similar yield-loss margin was reported by [12] in their field-based study using tropical maize germplasm. This study tested Al-stress tolerance at the seedling stage to identify promising lines early. Early growth stage selection helps breeders to accelerate genetic gain. It has been shown that the performance of maize under stress at the reproductive stage can be projected from its performance at the seedling and vegetative stages using growth traits such as plant biomass [16,17,18]. Thus, the current study is a reliable preliminary testing and stability analysis of elite QPM maize inbred lines for Al tolerance before advancing the best lines for further field testing. The findings of this study may be useful to fellow crop scientists, plant breeders and students of plant breeding and crop physiology.

## 4. Materials and Methods

### 4.1. Site Description

The study was carried out at the University of Fort Hare (UFH) in Alice, South Africa, once in the laboratory and twice during the 2019/2020 seasons in the greenhouse tunnel. The laboratory is in the Agronomy department under the Plant Breeding and Genetics Unit, and the greenhouse tunnel is at the University Farm. Light was provided by fluorescent lights (T5) that emit a 400–700-nanometer (nm) wavelength in the laboratory. The greenhouse tunnel was made up of clear polyethylene plastic that allows the transmission of sunlight. The average temperature in the greenhouse was 22 °C and 25 °C, respectively, for the two seasons, and 25 °C in the laboratory. Each trial in the laboratory and greenhouse was accompanied by its control counterparts, which received optimum management. Each trial was considered as an environment. Hence, there were six environments, which are the laboratory stressed with Al (LS), laboratory optimum (LC), first greenhouse stressed with Al (S1), first greenhouse optimum (C1), second greenhouse stressed with Al (S2) and second greenhouse optimum (C2).

### 4.2. Germplasm

A total of 75 quality protein maize (QPM) inbred lines were used in this study (Table 5), four of which were Al tolerant checks. The lines were obtained from CIMMYT-Zimbabwe, and Quality Seeds Company (Pvt, Ltd.) in KwaZulu-Natal, South Africa. CIMMYT-Mexico supplied four reference checks.

### 4.3. Experimental Procedures

#### Laboratory Experiment under Nutrient Solution

Quality protein maize seeds were surface sterilized with 0.1% sodium hypochlorite solution for 5 min by shaking continuously. The seeds were then thoroughly washed with tap water for another 5 min and rinsed with distilled water three times. This was followed by placing the seeds in petri dishes with moistened filter paper and germinating them at 27 °C for 7 days in an incubator. Five healthy and uniform germinated seeds per genotype were placed in plastic trays containing Hoagland solution for 3 days (72 h) at room temperature. The seedlings were grown for 72 h in the nutrient solution, after which they were transferred to the stress solution amended with an aluminum concentration level of 600 µM, while the control did not receive any aluminum sulfate (Al2 [SO4]3).

Five uniform seedlings per genotype were used for data collection, and each treatment was replicated three times in a completely randomized design (CRD). The Al concentration used in this study was adopted from [22], who reported that with 600 µM of ionic strength in the nutrient solution, the aluminum concentration would adequately discriminate aluminum-sensitive and aluminum-tolerant genotypes. A 1M HCl solution was used to adjust the pH of the stress solution to 4.0. The pH remained stable during the whole experiment without further adjustment. According to [30], a pH of 4 is important to activate Al toxicity, since the availability of most toxic forms of Al (Al^3+^) are dependent on the pH of the solution. The control solution had a pH of 5.8, which is optimum for seedling growth. The seedlings were harvested after the 72 h in stress conditions and weighed for dry matter determination.

### 4.4. Greenhouse Tunnel Experiment under Soil Bioassay

The experiment was laid out in a randomized complete block design (RCBD) with three replications in three blocks. The experimental procedure was adopted from [14] where alluvial soils, which were classified as Haplic Cambisol in accordance with the World Reference Base for Soil Resources (WRB) system [30] (pH 5.8), were collected from the UFH farm. The soil was watered to field capacity and incubated with Al sulphate (Al_2_ [SO_4_]_3_) for 48 h before planting. A pressure plate apparatus was used to determine the field capacity of the soil [31]. Aluminum sulphate (Al_2_ [SO_4_]_3_) was applied at the rate of 24 mg kg^−1^ of soil because the critical concentration of exchangeable cations above which toxicity is observed in the soil for most cereals is 23–24 mg/kg^−1^ [32]. At planting, plastic pots measuring 30 cm high and 11 cm wide were filled with soil amended with a compound basal fertilizer, hygrofert (153 g/kg N, 69 g/kg P, 183 g/kg Mg and 14 g/kg S). Two seeds per pot were sown and later thinned to one immediately after crop emergence. Hand pulling practice was performed for weed control, and no disease or pest incidence was observed. The pots were watered regularly to ensure that there was no moisture stress. The irrigation solution was maintained at pH 4.5 using HCl so that the Al^3+^ in the soil would be available to the plant throughout the experiment.

### 4.5. Data Collection and Analysis

In the laboratory the plants were harvested at 3 days after exposure to Al stress, and in the greenhouse tunnel 4 weeks after planting. This was followed by oven-drying the harvested plants at 65 °C until a constant weight was reached. The dry weight was determined by weighing the samples using a Microgram balance manufactured in Germany (d = 0.1 mg, Sartorius AG Gottingen CP 64).

### 4.6. Statistical Analysis

Statistical analyses were performed using GenStat^®^ 17th edition statistical software. A combined analysis of variance (ANOVA) across the environments allowed an estimation of the differences between the main effects (G), environment (E) and their interactions (G × E) on dry matter yield. To determine the stability values, the data on dry matter yield were further subjected to an AMMI analysis. A GGE biplot was used to assess the discriminating power of testing environments.

## 5. Conclusions

Our study has shown that the set of 75 QPM inbred lines used have genetic variation for Al tolerance. Hence, it is feasible to apply selection to improve dry matter yield under Al toxic and optimum growing conditions. However, more sources of variation should be introduced since the level of observed genetic variation was not very high. It was also revealed that greenhouse-based environments have better discriminating ability for Al tolerance and susceptibility than laboratory-based environments. Seventeen experimental lines were identified to possess a desired combination of high dry matter yield above the grand mean and ASVs that are less than the grand mean. They are therefore recommended for advancement to the field-testing stage. These are QSW 26, QSY 3, IBL 20, QSY 23, IBL4, IBL12, QSY 28, QSY16, QSW2, QSW 25, QSW 21, QSW 12, QSW 7, IBL17, IBL 21, IBL 15 and IBL 16. The findings of the study may be applied by maize breeders in their breeding programs.

## Figures and Tables

**Table 1:** Analysis of variance for dry matter yield of 75 QPM genotypes tested in six controlled environments.

Source of Variation	DF.	MS	Variation Contribution (%)
Environment	5	61,086.27 ***	82.82
Environment (Rep)	12	0 ^ns^	
Genotype	74	4483.23 ***	6.08
Genotype × Environment	370	8174.48 ***	11.08
Residual	888	15	0.02
Total	1349	73,758.98	

Environment: Al stress and non-stress experimental trials in the laboratory and greenhouse tunnel; DF: degrees of freedom; MS: mean squares; ***: significant at *p* < 0.0001; ^ns^: non-significant at *p* > 0.05.

**Table 2:** Additive main effects and multiplicative interaction analysis of variance for dry matter yield of 75 QPM genotypes tested across six environments.

Source of Variation	DF	M.S	Total Variation %	G × E Explained %
Total	1349	261		
Treatments	449	755 ***		
Rep	12	0 ^ns^		
Genotypes	74	4483.23 ***	6.08	
Environments	5	61,086.27 ***	82.82	
Interactions	370	8174.48 ***	11.08	
IPCA 1	78	4493 ***		54.64
IPCA 2	76	2654 ***		32.46689698
Residuals	216	17		
Error	888	15		

***: significant at *p* < 0.0001; ^ns^: not significant.

**Table 3:** Means and ASV of the top ten and bottom five yielding genotypes across the six environments. The genotypes are ranked according to mean dry weight (DW).

Genotype	Environments						Mean DW (g)	ASV
	C1	C2	LC	LS	S1	S2		
QSY 2	42.9	41.5	0.5	0.1	25.2	33.2	23.9	1.751
IBL 5	36.5	56.0	0.1	0.1	18.2	28.0	23.2	3.783
IBL 9	38.5	41.0	0.2	0.1	26.5	31.6	23.0	1.654
QSW 30	37.9	58.9	0.2	0.1	18.7	20.3	22.7	4.312
QSW 26	39.6	44.6	0.2	0.1	29.3	18.2	22.0	1.115
QSY 3	41.0	42.8	0.1	0.1	20.7	26.6	21.9	1.441
IBL 20	43.1	38.6	0.2	0.1	31.2	17.9	21.8	0.339
IBL 13	38.7	37.9	0.2	0.2	22.1	31.8	21.8	1.825
QSY 23	36.9	37.0	0.4	0.1	28.3	28.0	21.8	1.329
IBL 4	38.6	34.1	0.2	0.2	30.5	27.0	21.8	1.574
Top two high yielding checks								
CML 486	39.5	33.4	0.3	0.3	25.2	32.6	21.9	2.226
CML 435	37.4	37.1	0.1	0.1	23.9	27.1	21.0	1.244
Bottom three yielding checks								
QSY 20	31.9	32.3	0.1	0.0	19.0	12.7	16.0	0.257
QSY 24	36.5	22.3	0.6	0.1	20.7	15.5	16.0	2.077
QSY 9	31.0	36.0	0.2	0.1	12.9	14.1	15.7	1.135
QSY 21	30.6	40.1	0.8	0.1	14.4	5.9	15.3	2.363
QSY 12	35.0	29.2	0.8	0.2	16.4	7.6	14.9	0.897
Average	37.8	35.7	0.4	0.1	23.6	17.2	19.1	1.643

S1: first Al toxic environment under soil bioassay conditions in the greenhouse; S2: second Al toxic environment under soil bioassay conditions in the greenhouse; C1: first optimum environment under soil bioassay conditions in the greenhouse; C2: second optimum environment under soil bioassay conditions in the greenhouse; LC: optimum environment under nutrient solution conditions in the laboratory, LS: Al toxic environment under nutrient solution conditions in the laboratory; Mean DW(g): average dry weight in grams; ASV: AMMI stability value.

**Table 4:** Best four genotypes in each of the six environments.

Environment	Mean DW (g)	1	2	3	4
LS	0.1	IBL 20	QSY 5	QSW 7	QSW 26
LC	0.41	IBL 20	QSY 5	QSW 7	QSW 26
S1	23.57	QSY 14	QSW 17	IBL 20	QSY 16
S2	17.18	QSY 2	CML 486	IBL 13	IBL 9
C1	37.77	IBL 20	QSY 14	QSW 7	QSW 17
C2	35.69	IBL 8	QSW 30	IBL 5	QSY 27

S1: first Al toxic environment under soil bioassay conditions in the greenhouse; S2: second Al toxic environment under soil bioassay conditions in the greenhouse; C1: first optimum environment under soil bioassay conditions in the greenhouse; C2: second optimum environment under soil bioassay conditions in the greenhouse; LC: optimum environment under nutrient solution conditions in the laboratory, LS: Al toxic environment under nutrient solution conditions in the laboratory; Mean DW(g): average dry weight in grams.

**Table 5:** Names and sources 75 QPM germplasm used in this study.

Quality Seed Services (Pvt Ltd., South Africa) Germplasm					
Genotype Code Name	Genotype Full Name	Genotype Code Name	Genotype Full Name	Genotype Code Name	Genotype Full Name
QSY 1	D0620Y	QSY 24	EM578Y	QSW 14	HM18W
QSY 3	K0315Y	QSY 27	HM46Y	QSW 15	HM233W
QSY 5	S0181Y	QSY 28	HM48YE	QSW 16	HM238W
QSY 7	S0825Y	QSY 29	HM267Y	QSW 17	HM267W
QSY 8	T01292Y	29 E	HM260Y	QSW 18	HM267W
QSY 9	H0668Y	QSW 1	K054W	QSW 19	HM268W
QSY 10	V0377Y	QSW 2	S0181W	QSW 20	HM284W
QSY 12	A0595Y	QSW 3	S0507W	QSW 21	HM1472W
QSY 13	B0445Y	QSW 4	H0548W	QSW 23	JM234W
QSY 14	CM132Y	QSW 5	V0298W	QSW 24	JM2521W
QSY 16	CM231Y	QSW 6	B0388W	QSW 25	JM2561W
QSY 17	EM86Y	QSW 7	EM362W	QSW 26	JM2602W
QSY 18	EM88Y	QSW 8	EM583W	QSW 27	JM2621W
QSY 19	EM93Y	QSW 9	EM622W	QSW 28	JM2641W
QSY 20	EM114Y	QSW 10	EM625W	QSW 29	E5
QSY 21	EM130Y	QSW 12	GM15W	QSW 30	E6
QSY 23	EM560Y	QSW 13	GM44W	QSW 32	E27
CIMMYT-Zimbabwe germplasm					
Genotype code name	Pedigree				
IBL 1	CLQRCWQ50/CML312SR)-2-2-1-BB-1-B-B				
IBL 2	[[CML202/CML144] F2-1-1-3-B-1-B*6/[GQL5/[GQL5/[MSRXPOOL9]C1F2-205-1(OSU23i)-5-3-X-X-1-BB] F2-4sx]-11-3-1-1-B*4]-B*5-1-B				
IBL 5	[CML144/SNSYNF2[N3/TUX-A-90]-102-1-2-2-BSR-B*4]-B-4-3-B*4-1-B				
IBL 6	[CML150/CML373]-B-2-2-B*4-4-B-B				
IBL 7	[CML159/[CML159/[MSRXPOOL9] CIF2-205-1(OSU23i)-5-3-X-X-1-BB] F2-3sx]-8-1-1-BBB-4-B-B				
IBL 8	[CML182/TZMI703]-B-9-1-BB-#-BB-2-B-B				
IBL 9	[CML202/CML144] F2-1-1-3-B-1-B*6-2-B				
IBL 10	[CML205/CML176]-B-2-1-1-2-B*5-1-B-B				
IBL 11	[CML389/CML176]-B-29-2-2-B*4-B				
IBL 12	[GQL5/[GQL5/[MSRXPOOL9] CIF2-205-1(OSU23i)-5-3-X-X-1-BB] F2-4sk]-11-3-1-1-B*5-3-B-B				
IBL 13	[GQL5/GQL5/CML202] F2-3sk]-11-4-1-3-B*4-B				
IBL 14	[TZMI703/CML176]-B-3-2-B*5-4-B-B				
IBL 15	CLQRCWQ50-BB-1-2-B-B				
1BL 16	CML176-#-B-2-B				
IBL 17	CML181-B-1-5-B-B				
IBL 18	CML182-BB-B				
IBL 20	CML491-B-3-11-B-B				
IBL 21	CML492-BB-2-1-B-B				
IBL 22	WWO1408-1-1-2-B*4-#-B-B-B				
CIMMYT-Mexico Germplasm					
CML 304 *	-				
CML 435 *	-				
CML 486 *	-				
CML 439 *	-				

*: Al toxicity tolerant.

## Data Availability

Data will be availed when requested.

## Appendix A

**Table A1:** Mean yield all the 75 QPM maize inbred lines tested across the six environments.

Genotype	C1	C2	LC	LS	S1	S2	Mean DW	Stability (ASV)
QSY 2	42.9	41.5	0.5	0.1	25.2	33.2	23.9	1.751
IBL 5	36.5	56.0	0.1	0.1	18.2	28.0	23.2	3.783
IBL 9	38.5	41.0	0.2	0.1	26.5	31.6	23.0	1.654
QSW 30	37.9	58.9	0.2	0.1	18.7	20.3	22.7	4.312
QSW 26	39.6	44.6	0.2	0.1	29.3	18.2	22.0	1.115
CML 486	39.5	33.4	0.3	0.3	25.2	32.6	21.9	2.226
QSY 3	41.0	42.8	0.1	0.1	20.7	26.6	21.9	1.441
IBL 20	43.1	38.6	0.2	0.1	31.2	17.9	21.8	0.339
IBL 13	38.7	37.9	0.2	0.2	22.1	31.8	21.8	1.825
QSY 23	36.9	37.0	0.4	0.1	28.3	28.0	21.8	1.329
IBL 4	38.6	34.1	0.2	0.2	30.5	27.0	21.8	1.574
IBL 12	44.3	37.9	0.2	0.1	25.8	22.3	21.8	0.441
QSY 28	39.7	42.1	0.1	0.1	25.0	21.5	21.4	0.884
QSY 5	42.6	48.8	1.1	0.0	26.3	8.8	21.3	2.714
IBL 8	35.6	59.3	0.1	0.1	15.8	16.4	21.2	4.816
QSY 16	46.2	32.0	0.6	0.1	24.5	23.6	21.2	1.428
QSW 32	33.1	36.4	1.0	0.1	24.8	31.3	21.1	1.867
CML 435	37.4	37.1	0.1	0.1	23.9	27.1	21.0	1.244
QSW 2	38.4	42.9	1.9	0.1	24.9	16.8	20.8	1.187
IBL 6	34.7	49.0	0.1	0.1	21.4	19.0	20.7	2.56
QSW 14	37.6	46.6	0.1	0.1	25.1	14.1	20.6	2.052
QSW 25	40.2	33.3	0.2	0.1	28.7	21.0	20.6	1.056
QSY 14	42.1	28.5	0.1	0.1	29.6	22.9	20.6	2.135
QSW 16	39.0	30.2	0.2	0.2	25.7	27.3	20.4	2.011
QSW 21	40.2	36.6	0.1	0.1	30.2	15.1	20.4	0.565
QSW 12	37.1	34.0	1.1	0.0	21.0	27.6	20.2	1.492
QSY 27	37.4	49.7	0.5	0.1	24.0	9.0	20.1	3.056
QSW 7	38.1	37.5	0.2	0.1	33.3	11.0	20.0	1.048
QSW 17	42.9	27.2	0.2	0.1	28.1	20.9	19.9	2.173
IBL 17	41.4	35.5	0.1	0.1	20.2	22.1	19.9	0.635
IBL 21	35.2	42.1	0.1	0.1	24.5	17.5	19.9	1.17
IBL 15	40.3	41.9	0.2	0.1	23.3	13.2	19.8	1.348
QSW 1	37.9	43.7	0.2	0.1	25.9	11.0	19.8	1.787
QSW 29	39.7	28.1	0.2	0.1	28.4	22.0	19.8	1.997
QSW 6	38.7	45.0	0.7	0.1	22.0	11.0	19.6	2.168
IBL 16	35.0	41.4	0.1	0.1	20.0	20.1	19.4	1.323
QSY 29	39.2	27.2	0.2	0.2	25.3	24.0	19.4	2.145
IBL 1	41.6	30.6	0.1	0.1	20.8	20.0	18.9	1.061
QSW 28	38.1	33.6	0.2	0.1	22.8	18.2	18.8	0.41
CML 304	37.9	25.9	0.2	0.2	28.1	20.3	18.8	2.201
QSY 8	39.5	31.1	0.3	0.2	29.5	11.6	18.7	1.326
QSY 17	32.0	31.2	0.6	0.1	24.6	23.0	18.6	1.269
IBL 11	36.4	36.4	0.8	0.0	22.3	15.4	18.6	0.37
IBL 22	38.6	29.0	0.2	0.2	27.0	15.9	18.5	1.4
QSW 20	39.7	33.3	0.2	0.1	28.0	9.7	18.5	1.162
CML 439	35.7	23.7	0.2	0.1	27.8	23.0	18.4	2.695
IBL 7	35.5	42.8	0.2	0.1	18.7	12.9	18.4	1.895
IBL 14	40.0	34.4	0.2	0.0	23.6	11.0	18.2	0.805
QSW 10	32.9	43.6	0.2	0.1	16.7	15.2	18.1	2.117
QSW 3	35.9	36.6	0.1	0.1	24.2	11.6	18.1	0.792
QSW 19	31.8	39.0	0.2	0.2	23.8	13.0	18.0	1.054
IBL 2	35.9	24.6	0.1	0.1	26.7	19.4	17.8	2.222
QSY 10	36.7	40.1	0.1	0.0	16.9	11.8	17.6	1.571
IBL 10	42.5	28.0	1.9	0.1	25.8	7.0	17.5	1.909
QSW 18	37.7	25.5	0.1	0.1	22.5	18.8	17.5	1.839
QSW 4	41.7	35.3	0.9	0.0	16.8	9.6	17.4	1.035
QSW 15	37.6	22.8	0.2	0.2	27.3	16.0	17.4	2.475
QSY 18	39.2	31.2	1.9	0.1	24.7	6.8	17.3	1.454
QSW 8	33.4	32.1	0.1	0.1	18.7	18.0	17.1	0.438
IBL 18	41.6	21.7	1.0	0.1	21.1	16.7	17.0	2.459
QSW 9	32.5	36.3	0.2	0.1	23.5	9.6	17.0	1.023
QSW 24	32.4	29.0	0.2	0.1	23.5	15.7	16.8	0.921
QSW 13	39.6	28.0	1.8	0.0	25.7	4.6	16.6	1.949
QSW 27	36.8	22.4	2.0	0.1	21.7	16.4	16.6	2.196
QSW 5	37.5	31.7	0.2	0.1	22.1	7.2	16.5	1.162
QSW 23	33.1	36.3	0.2	0.1	19.1	10.0	16.5	1.098
QSY 7	37.2	21.5	0.1	0.1	24.8	14.7	16.4	2.478
29E	35.6	39.5	0.2	0.1	17.0	4.6	16.2	2.2
QSY 13	39.2	26.6	1.1	0.1	21.2	8.8	16.2	1.506
QSY 19	34.3	33.1	0.6	0.1	23.4	4.7	16.1	1.449
QSY 20	31.9	32.3	0.1	0.0	19.0	12.7	16.0	0.257
QSY 24	36.5	22.3	0.6	0.1	20.7	15.5	16.0	2.077
QSY 9	31.0	36.0	0.2	0.1	12.9	14.1	15.7	1.135
QSY 21	30.6	40.1	0.8	0.1	14.4	5.9	15.3	2.363
QSY 12	35.0	29.2	0.8	0.2	16.4	7.6	14.9	0.897
